# P-492. Immune Suppression and Anemia as Predictors of Opportunistic Infections in Children Living with HIV in the Dominican Republic

**DOI:** 10.1093/ofid/ofaf695.707

**Published:** 2026-01-11

**Authors:** Isabel Cintron, Carmen A Duluc, Maria Laura Andino, Laura P Mendez-Reyes, Claudio Andujar, Caridad Abud, Karla Tavarez, Ana Lia Flavia, Daliah Rodriguez, Rosa Abreu, Milagros Peña, Hector Jose Lora-Rodriguez, Robert Paulino-Ramirez

**Affiliations:** Hospital Pediátrico Dr. Robert Reid Cabral, Santo Domingo, Distrito Nacional, Dominican Republic; Universidad Iberoamericana, Santo Domingo, Distrito Nacional, Dominican Republic; Universidad Iberoamericana, Santo Domingo, Distrito Nacional, Dominican Republic; Universidad Iberoamericana, Santo Domingo, Distrito Nacional, Dominican Republic; Universidad Iberoamericana, Santo Domingo, Distrito Nacional, Dominican Republic; Universidad Iberoamericana, Santo Domingo, Distrito Nacional, Dominican Republic; Universidad Iberoamericana, Santo Domingo, Distrito Nacional, Dominican Republic; Universidad Iberoamericana, Santo Domingo, Distrito Nacional, Dominican Republic; Universidad Iberoamericana, Santo Domingo, Distrito Nacional, Dominican Republic; Hospital Infantil Dr. Robert Reid Cabral, Santo Domingo, Distrito Nacional, Dominican Republic; Hospital Infantil Dr. Robert Reid Cabral, Santo Domingo, Distrito Nacional, Dominican Republic; Yale New Haven Hospital, Distrito Nacional, Distrito Nacional, Dominican Republic; Universidad Iberoamericana, Santo Domingo, Distrito Nacional, Dominican Republic

## Abstract

**Background:**

In the Dominican Republic (DR), an estimated of 810 children aged 0–14 years, were living with HIV in 2023. Opportunistic infections (OIs) remain a major contributor to morbidity among pediatric patients with HIV, yet there is a critical lack of localized research exploring the clinical and social determinants of severe infections. This study aimed to contextualize the clinical and sociodemographic factors associated with the development of OIs among children living with HIV in the DR.

**Figure:**
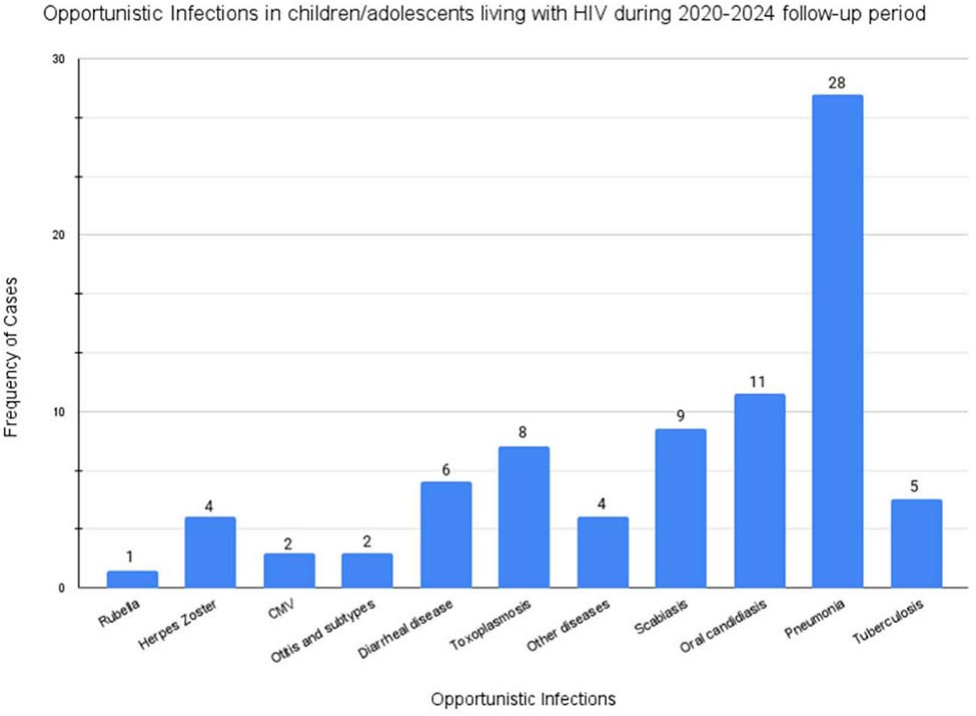

**Methods:**

A retrospective cohort analysis was conducted using clinical data from 79 children living with HIV (CLHIV) who received care between 2020 and 2024 at the HIV Comprehensive Care Center of the national pediatric referral hospital. Data extracted from medical records included demographic variables, route of HIV transmission, CD4 T-cell counts, viral load (VL), and other clinical parameters. Descriptive and statistical analyses were performed using JASP software.

**Results:**

Of the 79 pediatric patients analyzed, 54.43% were female, with a mean age of 89.97 months. 96.20% acquired HIV via vertical transmission. During the four-year study period, 70.89% developed at least one OI, with pneumonia (Figure 1) identified as the most common infection (35.44%). Statistical analysis revealed that lower CD4 counts were significantly associated with the occurrence of OIs (ANOVA: F(1,72) = 4.007, p = 0.049; Pearson’s r = -0.271, p = 0.019). Hemoglobin levels were also significantly lower among those with OIs (ANOVA: F(1,73) = 4.599, p = 0.035), suggesting an interplay between immune suppression, inflammatory processes, and possible nutritional deficiencies. No significant association was observed between current VL and the presence of OIs.

**Conclusion:**

This study underscores the substantial burden of OIs among CLHIV in the DR, highlighting pneumonia as the predominant infection. Lower CD4 counts and hemoglobin levels were significantly associated with the development of OIs, reflecting profound immune compromise. These findings emphasize the urgent need for enhanced pediatric HIV care, including strengthened prenatal HIV screening, earlier initiation of antiretroviral therapy, and robust longitudinal monitoring to mitigate the risk of OIs and improve clinical outcomes in this vulnerable population.

**Disclosures:**

All Authors: No reported disclosures

